# Deep-water fossorial shrimps from the Oligocene Kiscell Clay of Hungary: Taxonomy and palaeoecology

**DOI:** 10.4202/app.2012.0078

**Published:** 2014

**Authors:** MATÚŠ HYŽNÝ, ALFRÉD DULAI

**Affiliations:** [hyzny.matus@gmail.com], Department of Geology and Palaeontology, Natural History Museum, Burgring 7, Vienna 1010, Austria; Department of Geology and Palaeontology, Faculty of Natural Sciences, Comenius University, Mlynská dolina G1, Bratislava 842 15, Slovakia; [dulai@nhmus.hu], Department of Palaeontology and Geology, Hungarian Natural History Museum, Ludovika tér 2, Budapest H-1088, Hungary

**Keywords:** Decapoda, Callianassidae, *Lepidophthalmus*, Ctenochelidae, *Ctenocheles*, systematics, deep-water environment, Oligocene, Hungary

## Abstract

We describe deep-water ghost shrimp assemblages from the otherwise well known Oligocene Kiscell Clay in Hungary. The described fossorial shrimps (Decapoda: Callianassidae and Ctenochelidae) include: *Ctenocheles rupeliensis* (younger synonym *Callianassa nuda*) and *Lepidophthalmus crateriferus* (younger synonym *Callianassa brevimanus*). The fossil material of the former species is assigned to *Ctenocheles* based on the morphology of the major cheliped, particularly the pectinate fingers, bulbous propodus, cup-shaped carpus and elongated merus. *Lepidophthalmus crateriferus* from the Oligocene of Hungary is the first unequivocal fossil record of the genus, which is distinguished in the fossil record on the basis of the presence of a meral blade and meral hook on the major cheliped. *Lepidophthalmus* is today known exclusively from shallow-water environments. The finding of a deep-water fossil representative of *Lepidophthalmus* therefore appears to be a reverse of the common pattern of groups shifting environments from onshore to offshore over geological time, as seen in many taxa. The presence of *Lepidophthalmus crateriferus* comb. nov. in the Kiscell Clay therefore suggests different ecological requirements for at least some populations of this genus in the geological past.

## Introduction

The fossil record of deep-water decapod crustacean assemblages is poorly known and only a few of them have been reported so far (e.g., Beurlen 1939; Takeda et al. 1986; Feldmann et al. 1991; Karasawa 1991, 1993; Kato 1996; Charbonnier et al. 2010; Hyžný and Schlögl 2011). They’re often known from special cases such as hydrocarbon seeps and hydrothermal vents (Bishop and Williams 2000; Campbell 2006; Peckmann et al. 2007; Schweitzer and Feldmann 2008; Charbonnier et al. 2010; Karasawa 2011). Ghost shrimps (several families treated together as Callianassoidea Dana, 1852) in Recent environments constitute important elements of predominantly shallow intertidal and subtidal marine ecosystems, although several exclusively deep-water taxa are also known (Dworschak 2000, 2005). In Cenozoic assemblages, identified as coming from deep-water environments, callianassoid shrimps, specifically *Callianopsis* de Saint Laurent, 1973, were also present (Feldmann et al. 1991; Karasawa 1991, 1993; Kato 1996; Hyžný and Schlögl 2011). Beurlen (1939) described a conspicuous decapod fauna from the Kiscell Clay, Hungary consisting of several taxa (Table 1). Ghost shrimps constitute its most abundant component, with *Ctenocheles rupeliensis* (Beurlen, 1939) representing one of the most common macrofossils of the typical Kiscell Clay assemblage (Báldi 1986).

The aim of the paper is to taxonomically redescribe the Oligocene (Rupelian) ghost shrimp fauna of the Kiscell Clay based both on the original material of Beurlen (1939) and additional collections, and to discuss its palaeoecological implications. This material allows *Callianassa nuda* Beurlen, 1939 to be synonymized with *C. rupeliensis*, and *C. brevimanus* Beurlen, 1939 to be synonymized with *C. craterifera* Lörenthey in Lörenthey and Beurlen, 1929. Subsequently, the latter taxon is reassigned to *Lepidophthalmus* Holmes, 1904, thus representing the first unequivocal fossil record of this genus. The Kiscell Clay decapod fauna clearly represents a deep-water assemblage whose environmental requirements can be correlated with other faunal elements; i.e., foraminifers, corals, brachiopods, bivalves, gastropods, ostracods, cirripedes, and fishes.

### Institutional abbreviations

FI, Hungarian Geological and Geophysical Institute (Magyar Földtani és Geofizikai Intézet) in Budapest, Hungary; HNHM, Department of Paleontology and Geology, Hungarian Natural History Museum in Budapest, Hungary; KGP-MH, Department of Geology and Palaeontology, Comenius University in Bratislava, Slovakia; NHMW, Natural History Museum in Vienna, Austria.

## Geological and geographical setting

### General remarks on the geology of the area

The Paratethys was an epicontinental sea that formed in the Early Oligocene as a consequence of Africa’s northward movement and the resulting subduction of the European plate (Báldi 1980). It was intermittently connected to the Mediterranean and the Indo-Pacific (Rögl 1998, 1999; Harzhauser and Piller 2007; Harzhauser et al. 2007). The area from present-day Austria to Poland, Ukraine and Romania is called the Central Paratethys. The Kiscellian is a regional stage used in the Central Paratethys for part of the Lower Oligocene. It was first proposed (Báldi 1979), and later formally described by Báldi (1986). The Kiscellian corresponds to the Rupelian and the lowest part of the Chattian, while the overlying Egerian comprises the middle and upper part of the Chattian and the lower part of the Aquitanian (Báldi et al. 1999; Piller et al. 2007).

**Fig. 1:**
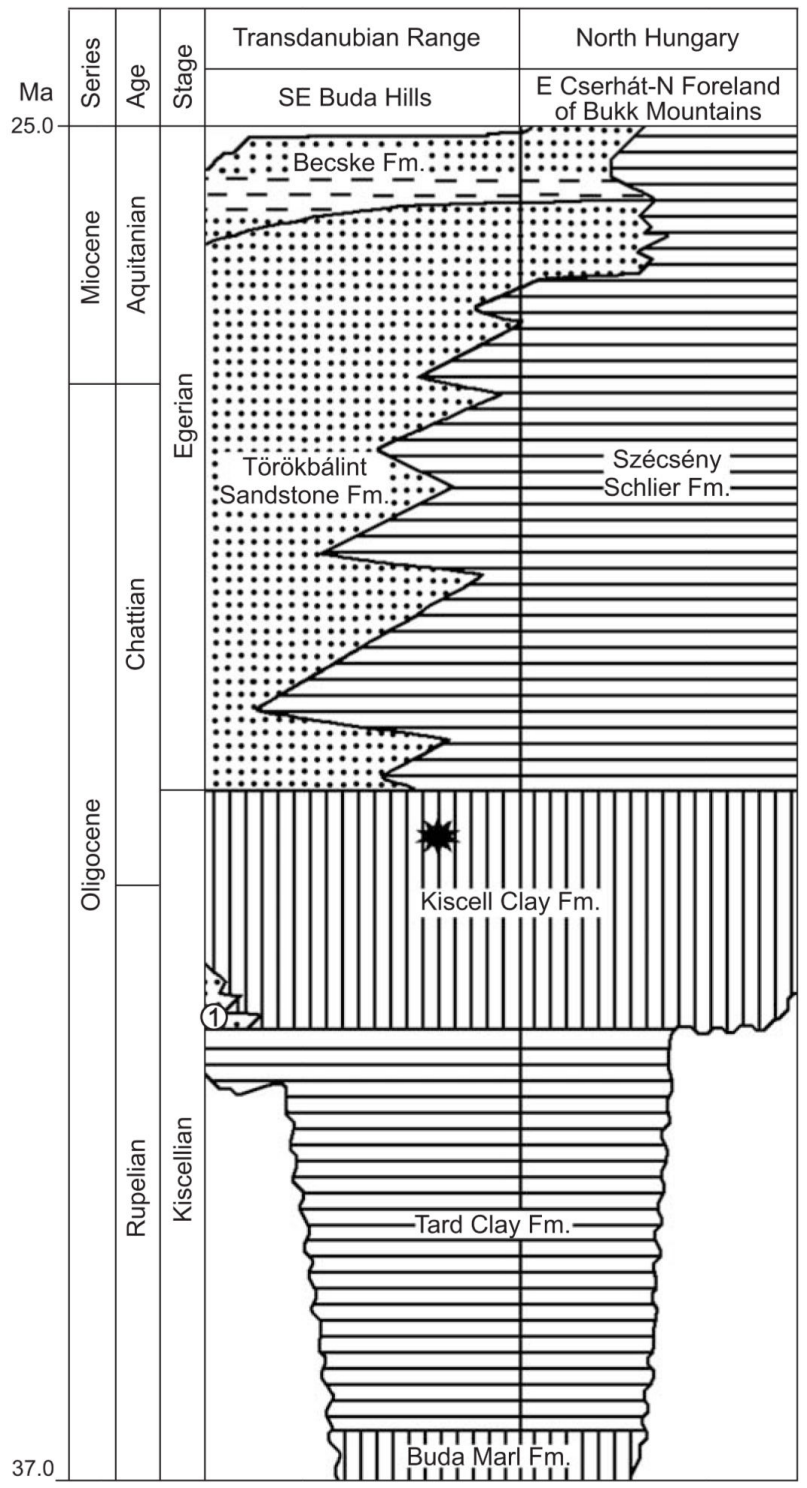
Lithostratigraphic units of the Hungarian Oligocene at the Buda Hills area (modified after Császár 1997). Asterisk, approximate position of the studied samples; 1, Hárshegy Sandstone Formation.

During the Oligocene the area of the Buda Mountains was part of the Hungarian Paleogene Basin. Although the larger part of the bathyal Buda Marl was deposited in the Late Eocene, calcareous nannoplankton and planktonic foraminiferan studies have revealed that its uppermost layers represent the lowermost Oligocene (NP 21–22 nannoplankton zones, P 18 plankton foraminifer zone; Nagymarosy 1992; Horváth 1998) (Fig. 1). At the beginning of the Oligocene the Central Paratethys was separated from the Mediterranean and laminated black shales were deposited in the anoxic environment of the restricted basin (Tard Clay Formation, “fish shale”) (Báldi 1984). This formation is generally poor in fossils. The age of the lower part of the Tard Clay was estimated to Early Kiscellian, P 18 foraminifera zone (Horváth 2002). The Kiscell Clay conformably overlies the Tard Clay. At the time of its deposition the connection with world oceans was restored and anoxia ceased (Báldi 1983, 1986). The name of the Kiscell Clay is derived from the Kiscell plateau located in the Buda Mountains. The Kiscell Clay consists of grey calcareous clay and clayey marl, which is not stratified or laminated but is well bioturbated (Báldi 1983).

Kiscell at Óbuda (northwestern part of Budapest) is the type area of the Kiscellian stage. In the second half of the 19th century remarkable building operations were carried out in Budapest area and the building material was mined in the brickyards of Óbuda. The most famous was the Újlak brickyard (former Holzspach brickyard), as this is the type locality of the formation and most fossils were collected there. Unfortunately, Óbuda is recently a densely populated residential area and the former brickyards disappeared or were recultivated. Therefore, the classical localities are not accessible any more. Nowadays, in the environs of Budapest, the Kiscell Clay is mined only at Pilisborosjenö and Törökbálint (Horváth 2002).

### Stratigraphy of the Kiscell Clay

The nannoflora of the Kiscell Clay belongs to the lower part of NP 24 zone (Late Kiscellian) (Nagymarosy and Báldi-Beke 1988). The lower stratigraphical level (lowermost 50–100 m) in the Kiscell Clay can be characterized by *Cassidulina vitalisi* Majzon, 1948 from the *Globigerina–Gemellides–Uvigerina* assemblage (Horváth 1998). The ratio between calcareous and agglutinated foraminifers is variable depending on the quantity of sandy sediment influx. This assemblage probably belongs to the topmost part of the P 20 and the lower part of the P 21 plankton foraminifera zones (Horváth 1998). In the upper part of the Kiscell Clay the relatively large-sized (1–5 mm) agglutinated taxa are dominant (Horváth 1998). The agglutinated specimens often amount up to 50% of the total foraminiferal fauna. Planktonic forms are rare or missing. This assemblage also belongs to the Late Kiscellian (NP 24 nannoplankton zone) and P 21 plankton foraminifera zone (Horváth 1998, 2002). K-Ar dating of the glauconite from the Kiscell Clay at Pilisborosjenö (north of Budapest) gives an age of 33+/−3 Ma (Báldi et al. 1975).

### Review of faunal elements of the Kiscell Clay

The Kiscell Clay is generally not very rich in macrofossils. Sediments of this formation, however, were mined in several brickyards along the rims of the Buda Mts for nearly 100 years and therefore its fauna is relatively well-known.

Nevertheless, the best known fossils in the Kiscell Clay are microfauna, and above all foraminifers which were first described in a classic monograph by Hantken (1875) as “*Clavulina Szabói* layers” (= upper part of the Buda Marl and the Kiscell Clay). Up to now, almost 500 species of foraminifers have been identified in the Kiscell Clay (Hantken 1875; Majzon 1966; Sztrákos 1974; Gellai-Nagy 1988). The preserved part of Hantken’s (1875) material was revised recently by Horváth (2002, 2003). Most of the foraminifers are benthic forms with a relatively slow evolutionary rate and their distribution was mainly affected by local environmental factors.

The Kiscell Clay contains a rich hemipelagic nannoflora (Nagymarosy and Báldi-Beke 1988). Dominating forms are placoliths, together with helicosphaerids and discoliths. Tropical elements, such as discoasterids, are completely missing (Nagymarosy and Báldi-Beke 1988).

The mollusc fauna of the Kiscell Clay (mostly collected at the Újlak brickyard) was monographically described by Noszky (1939, 1940). On the basis of very small and insignificant differences he recognized 764 forms in this fauna. After the revision of Noszky’s material, Báldi (1986) distinguished only 169 mollusc species (66 gastropods, 98 bivalves, 1 scaphopod, and at least 4 nautiloids).

Brachiopods are represented by *Terebratulina caputserpentis* (= *T. tenuistriata* [Leymerie, 1846]) whose presence at the Újlak brickyard was reported by Meznerics (1944).

The presence of echinoderms in the Kiscell Clay is questionable. Kolosváry (1941) described *Pseudaspidura hungarica* Kolosváry, 1941 as an ophiuroid; however, Kroh (2002) recently cast doubt on its ophiuroid affinity.

The fish fauna of the Kiscell Clay was studied by Weiler (1933, 1938) who identified several sharks and bony fishes. A rich otolith fauna (30 taxa) was described from the Kiscell Clay; however, this was not from the Budapest area but from the surroundings of Eger (Northeastern Hungary) by Nolf and Brzobohatý (1994). Marine mammals are represented by *Halitherium* Kaup, 1838 remains at the Újlak brickyard and about 30 cetacean vertebrae at the Farkasrét cemetery location (Kretzoi 1941).

Crustaceans of the Kiscell Clay are represented by several high-level taxa. The ostracod fauna is represented by *Cytherella compressa* (Münster, 1830), *C. dentifera* Méhes, 1941, *C. hyalina* Méhes, 1941, *Bairdia rupelica* Monostori, 1982, *Paijenborchella sturovensis* Brestenská, 1975, *Krithe pernoides* (Bornemann, 1855), *Parakrithe costatomarginata* Monostori, 1982, *Costa hermi* Witt, 1967, *Agrenocythere ordinate* (Deltel, 1961), and some others (see Monostori 1982, 2004). This composition shows that this assemblage is not typical for the Tard Clay fauna, but are rather a reminiscent of the fauna of the lowermost Oligocene beds (Monostori 2008). Cirripeds are represented by the bathyal genus *Scalpellum* Leach, 1818 which most probably cemented to swimming organisms post-mortem during their deposition in the deep-water sediments (Szörényi 1934).

A decapod crustacean fauna of the Kiscell Clay is represented by five species (Table 1). The only account of this fauna was published by Beurlen (1939) who described six new taxa; some of them are recognized as junior synonyms herein.

## Material and methods

The studied samples mostly consist of the material originally described by Beurlen (1939). Additional material comes from subsequent collecting by different workers and has not been previously reported in the literature. The material is preserved either three-dimensionally or partially compressed. Most samples are represented by isolated major chelae. In such cases the dactylus is usually still articulated with the propodus. Several samples exhibit preservation of both chelae and two specimens retain remains of the carapace and pleon. The matrix is rather soft, thus enabling easy preparation. To enhance contrast most material was coated with ammonium chloride prior to photography.

The studied material presented herein was thoroughly compared with published accounts (descriptions and figures) of fossil and extant callianassoid taxa. Additionally, comparative extant material was also studied, namely *Lepidophthalmus eiseni* Holmes, 1904 (NHMW 19790); *L. louisianensis* (Schmitt, 1935) (NHMW 6977); *L. richardi* Felder and Manning, 1997 (NHMW 25292); *L. sinuensis* Lemaitre and Rodrigues, 1991 (NHMW 25288); *L. siriboia* Felder and Rodrigues, 1993 (NHMW 6897); *L. tridentatus* (von Martens, 1868) (NHMW 18323); *L. turneranus* (White, 1861) (NHMW 6795, 18347); and *Ctenocheles maorianus* Powell, 1949 (NHMW 6733).

## Systematic palaeontology

### Order Decapoda Latreille, 1802 Infraorder Axiidea de Saint Laurent, 1979 Superfamily Callianassoidea Dana, 1852 Family Callianassidae Dana, 1852

#### Discussion

This long recognized family of fossorial shrimps has a robust fossil record consisting of 218 named species (Schweitzer et al. 2010) and spanning from the Early Cretaceous to Holocene. However, the evolutionary relationships between respective taxa are hindered as more than one-third of all species are classified within the waste-basket-taxon “*Callianassa*”. As a result, the callianassid fossil record is in need of revision. Unfortunately there are discrepancies in proposed biological classifications of the group (Manning and Felder 1991; Poore 1994; Sakai 1999b, 2005, 2011; De Grave et al. 2009). Relationships between genera are also not completely clear (cf. Tudge et al. 2000; Felder and Robles 2009; Robles et al. 2009; see also Dworschak et al. 2012). The assignment of fossil material to biologically defined genera was recently discussed by Schweitzer and Feldmann (2002), Schweitzer et al. (2006), Hyžný and Karasawa (2012), Hyžný and Hudáčková (2012) and Hyžný and Müller (2012).

### Subfamily Callichirinae Manning and Felder, 1991 Genus *Lepidophthalmus* Holmes, 1904

#### Type species

*Lepidophthalmus eiseni* Holmes, 1904, by monotypy; San Jose del Cabo, Lower California, Pacific.

#### Species included

*Lepidophthalmus crateriferus* (Lörenthey in Lörenthey and Beurlen, 1929) comb. nov. from the Oligocene of Hungary and several Recent species (see Poore 2012).

#### Emended diagnosis

Carapace with rostral spine; cornea dorsal, subterminal, disk-shaped; antennular peduncle longer and stouter than antennal peduncle; third maxilliped with minute exopod, ischium-merus subpediform, merus not projecting beyond articulation with carpus; chelipeds unequal, merus of major cheliped with meral hook positioned proximally and blade positioned distally; first pleopod slender and uniramous, second pleopod slender and biramous, third to fifth pleopods foliaceous and biramous in both sexes, appendices internae digitiform and distal on second pleopod, stubby, embedded in margin of endopod on third to fifth pleopods in both sexes (emended from Manning and Felder 1991: 778).

#### Discussion

*Lepidophthalmus* waas considered indistinguishable from *Callianassa* by de Man (1928) and Schmitt (1935). The genus was resurrected by Manning and Felder (1991) and it was treated as valid by subsequent authors (e.g., Poore 1994; Felder and Manning 1997; Sakai 1999b, 2005). Manning and Felder (1991) considered the type species (*L. eiseni*) a junior synonym of *L. bocourti* (A. Milne Edwards, 1870). Felder (2003) showed that both taxa are distinct. Sakai (2005) still treated *L. eiseni* as synonymous with *L. bocourti*. In his latest monograph, Sakai (2011) redefined the genus substantially; he considered both the above mentioned species as distinct and *L. bocourti* (assuming that it represents the type species) to be the only member of the genus. He erected a new genus *Lepidophthalmoides* with *L. eiseni* (!) as its type species for all other previously recognized *Lepidophthalmus* species. Therefore, *Lepidophthalmoides* is an objective junior synonym of *Lepidophthalmus* as both genera are based on the same type species. Thus, in treating *Lepidophthalmus* as valid we follow here Manning and Felder (1991), Felder (2003), and Poore (2012).

Species of *Lepidophthalmus* are strongly heterochelous. They usually possess a rather stout major cheliped which can be heavily armed, especially in large males.

The merus of the major cheliped always possesses a proximal hook, which is sometimes bifid (or trifid), and a distally positioned pronounced blade (or lobe). The blade usually possesses serrations or small teeth (e.g., Rodrigues 1971: figs. 29, 30; Felder and Rodrigues 1993: figs. 1d, 1e, 3b, 3c; Felder and Manning 1997: figs. 1b, 2h, 2i, 3a–c; Felder 2003: figs. 13, 22). It seems that the meral blade is already present in small specimens (Peter C. Dworschak, personal communication 2011) and therefore can be considered of taxonomic value for palaeontologists. In extant *Lepidophthalmus* species, the only exception is *L. socotrensis* Sakai and Apel, 2002, in which the merus has a broad lobate projection in larger males instead of a tiny medal hook (Sakai and Apel 2002: figs. 5c, 6a), and the lower margin, although serrated, does not possess any distal blade. In virtually all *Lepidophthalmus* species the upper margin of the merus is clearly convex and slightly or strongly concave proximally, sometimes forming a U-shaped notch near the articulation with the ischium (Sakai 1970: fig. 2a; Felder and Rodrigues 1993: fig. 4c; Felder and Manning 1997: figs. 1b, 2i, 3a; Dworschak 2007: figs. 11, 13). This notch is usually present on large males; thus, its development seems to be correlated with age, size and sex.

The carpus is semirectangular with the lower margin distinctly rounded proximally; the upper margin is slighthly converging proximally. The carpus is approximately as long as the palm, but differs in length between individuals. Holmes (1904) noted that in *L. eiseni* the carpus is somewhat shorter in males compared to that of females. A distinctly shorter carpus than palm was figured in both sexes for *L. rosae* (Nobili, 1904), *L. tridentatus* (von Martens, 1868), and *L. turneranus* (White, 1861) (Sakai 2005: figs. 31A–C; Dworschak 2007: figs. 2, 4–7, 11–14, 23–25, 32–35; de Saint Laurent and Le Loeuff 1979: figs. 20a, b; respectively).

The propodus is seemingly sexually dimorphic. Although no extensive study on sexual dimorphism within the chelipeds of *Lepidophthalmus* has been conducted so far (except for chela measurements, see Felder and Lovett 1989), thorough comparison of published figures and descriptions of all described species clearly shows that males usually have a propodal notch (sometimes termed as gape) with a distal tooth, both positioned just above the fixed finger at the articulation with the dactylus. There may also be a depression on the lateral and mesial surfaces of the palm positioned just between the fingers. This depression is usually well visible in low-angled light, and is usually covered with large tubercles. The depression can be large (up to half of the palm length) and is distinctly triangular in its shape. The depression in females normally is not present or is significantly reduced. Moreover, they have no notch between fingers; rather their fixed finger is broader than in males. Upper and lower margins of the propodus in females are distinctly converging distally; the lower margin can be broadly sinuous. These sexual differences in major cheliped morphology seem to be consistent within the genus, although a few exceptions can be found. In *L. turneranus* the above described male morphotype is present in females too, at least according to published figures (de Saint Laurent and Le Loeuff 1979: fig. 20b).

Virtually all *Lepidophthalmus* species have a keeled fixed finger, although this character is not always apparent during examination and may be obscured by compaction when preserved in the fossil state. In many extant species the fixed finger of males possesses a large triangular tooth on its occlusal margin, which can be directed distally (e.g., in *L. manningi*, see Felder and Staton 2000: fig. 1c; in *L. richardi*, see Felder and Manning 1997: figs. 4d–f; in *L. siriboia*, see Felder and Rodrigues 1993: fig. 4c; in *L. sinuensis*, see Lemaitre and Rodrigues 1991: figs. 3a, 3b). In males the dactylus is heavily armed with several teeth of different shapes depending on species. Females usually have unarmed dactyli, or at least the teeth are less developed than in males.

The minor cheliped is distinctly smaller than the major one and is usually unarmed. The merus is ovoid and may possess or lack a meral hook. The propodus is usually tapering distally and its lower margin is slightly concave at the articulation with the fixed finger. Both fingers are longer than the palm, and the dactylus is keeled.

As mentioned above, *Lepidophthalmus socotrensis* seems to be different from all other congeners. It has no tuberculation on the lateral surface of the propodus in the major cheliped, no notch or distal tooth on the distal margin at the base of the fixed finger and possesses a strongly armed minor cheliped dactylus. Also the sexual dimorphism in the nature of the major propodus as discussed above is not consistent within this species. As a result, we do not consider it a typical *Lepidophthalmus*. Indeed recently, Sakai et al. (2014) synonymized *L. socotrensis* with *Podocallichirus madagassus* (Lenz and Richters, 1881).

**Fig. 2:**
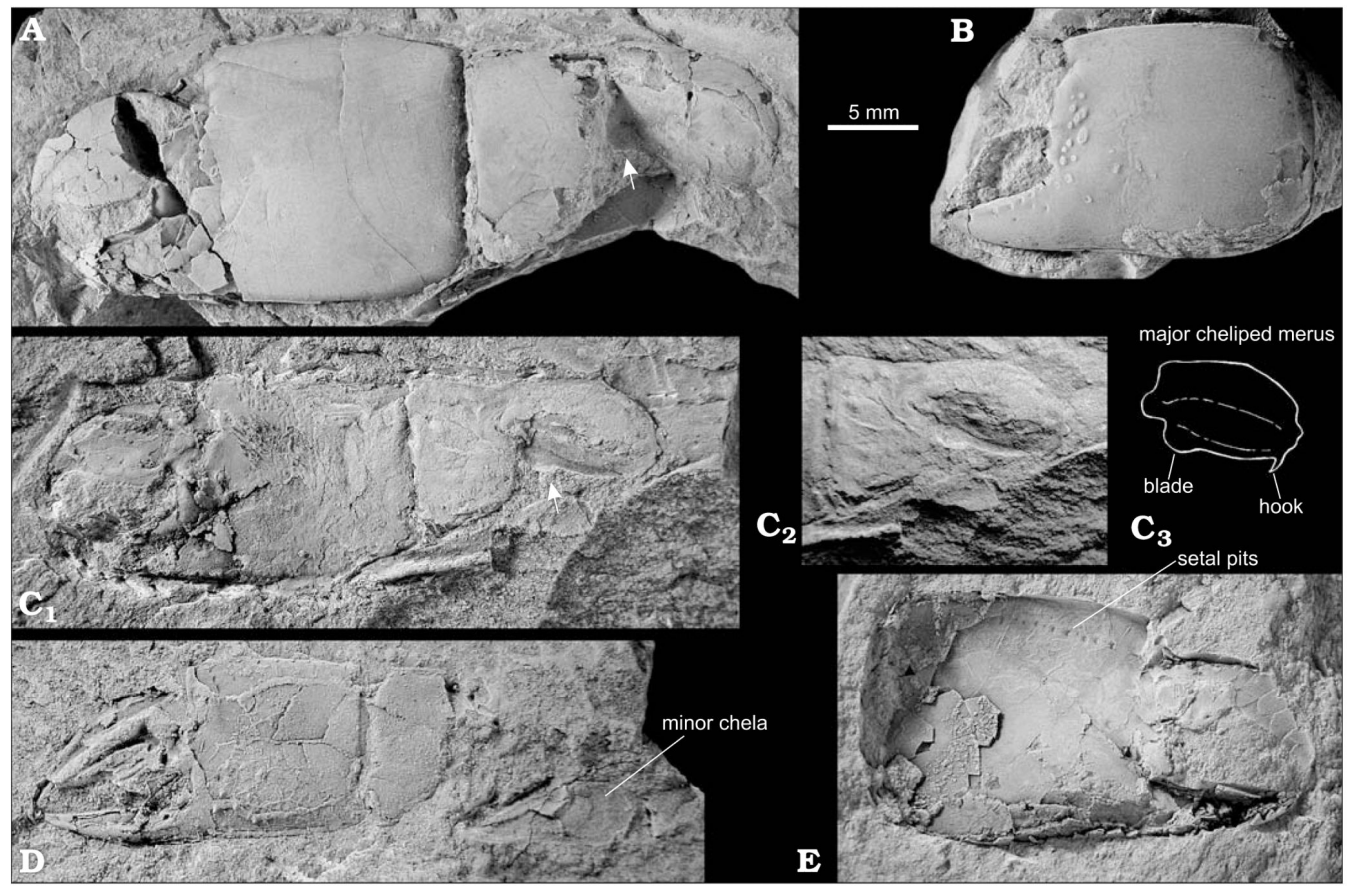
Fossorial shrimp *Lepidophthalmus crateriferus* (Lörenthey in Lörenthey and Beurlen, 1929) comb. nov., Óbuda in Budapest, Late Kiscellian. **A**. Left major cheliped of presumed male (HNHM M.59.4684b). **B**. Isolated left major propodus (HNHM M.59.4690). **C**. Left major cheliped of presumed male (C_1_); neotype herein designated (lectotype of *Callianassa brevimanus* Beurlen, 1939) (HNHM M.59.4684a). Detail of C_1_ under different light angle showing carpus and merus (C_2_). Line drawing of merus depicted in C_2_ (C_3_). Note presence of distal meral hook and blade (see also white arrows in A and C_1_). **D**. Presumed female specimen with both chelae (HNHM M.59.4720). **E**. Imprint of mesial surface of right major propodus (HNHM M.59.4683). Note setal pits close to upper margin of the chela. All specimens except HNHM M.59.4684a are paralectotypes of *C. brevimanus* selected herein. All specimens are figured to the same scale and were covered with ammonium chloride (except C_2_) prior to photography. Photographs by MH.

Manning and Felder (1991) pointed out the taxonomic importance of the merus on the major cheliped, usually in combination with other characters, as a distinctive feature for the generic assignment of ghost shrimps. The meral hook is present in many callianassoid taxa (mostly in the subfamily Callianassinae); its development, however, is strongly variable among different genera and in many cases it can help in taxonomic determination. A tiny meral hook in its distal position is present in several genera, although, only *Lepidophthalmus* and *Callianopsis* de Saint Laurent, 1973 can be compared to each other as both share rather similar morphology of cheliped elements. In both taxa the general outline of the merus is similar, but contrary to *Lepidophthalmus, Callianopsis* does not possess a distal meral blade, the proximal meral hook is never bifid and the upper margin has no distinct proximal concavity (Schweitzer Hopkins and Feldmann 1997: fig. 4A, B; Lin et al. 2007: fig. 1C). Both genera otherwise share similarly shaped major propodus in males and females and possession of tubercles on its lateral surface. Males of *Lepidophthalmus* species may have a large triangular tooth on the occlusal margin of the fixed finger which is present also in *Callianopsis goniophthalma* (Rathbun, 1902) (Schweitzer Hopkins and Feldmann 1997: fig. 4A). Major distinctions between both genera lie in the presence of a propodal depression in *Lepidophthalmus*, which is missing in *Callianopsis*. There may be a distinction in the nature of the carpus which seems to be always shorter than the propodus in *Callianopsis* but in *Lepidophthalmus* its length greatly varies and is at least partially dependent on sex. Males usually have a shorter carpus; in females it is at least as long as the palm. The shape of the minor cheliped of both genera is also strikingly different; *Callianopsis* has a sharp distally oriented tooth situated on the occlusal margin of the fixed finger (Schweitzer Hopkins and Feldmann 1997: fig. 4C; Lin et al. 2007: fig. 1D; Hyžný and Schlögl 2011: text-figs. 2A, B, E, F), which *Lepidophthalmus* lacks.

Neontologists rely on the soft part morphology to identify callianassid taxa, which is usually not present in the fossil record. Therefore, the distinctive shape of the merus as discussed above (tiny meral hook and presence of meral blade) can be convincingly used as a proxy character for the generic assignment of fossil material to *Lepidophthalmus*. The meral hook in *Lepidophthalmus* is often bifid or even trifid, but due to compaction and general imperfection of preservation in the sedimentological record this morphological feature may be obscured. We propose that the distal meral blade can be considered of taxonomic importance in distinguishing the genera discussed here. The merus in *Lepidophthalmus* is also somewhat deeper in comparison with *Callianopsis*, although this feature may be a matter of preservation. In this respect the generic assignment of *Callianopsis australis* Casadío, De Angeli, Feldmann, Garassino, Hetler, Parras, and Schweitzer, 2004 from the middle Oligocene of Argentina (Casadío et al. 2004) and *C. inornatus* Schweitzer and Feldmann, 2001 from the Eocene of Washington, USA (Schweitzer and Feldmann 2001) may be revisited as the merus in these taxa is distinctly ovoid, a shape not commonly seen in this genus (compare Schweitzer Hopkins and Feldmann 1997). On the other hand, the overall morphology of *C. inornatus* chelipeds (Schweitzer and Feldmann 2001: fig. 9.3) clearly excludes the possibility of identifying this taxon as a member of *Lepidophthalmus*.

**Fig. 3:**
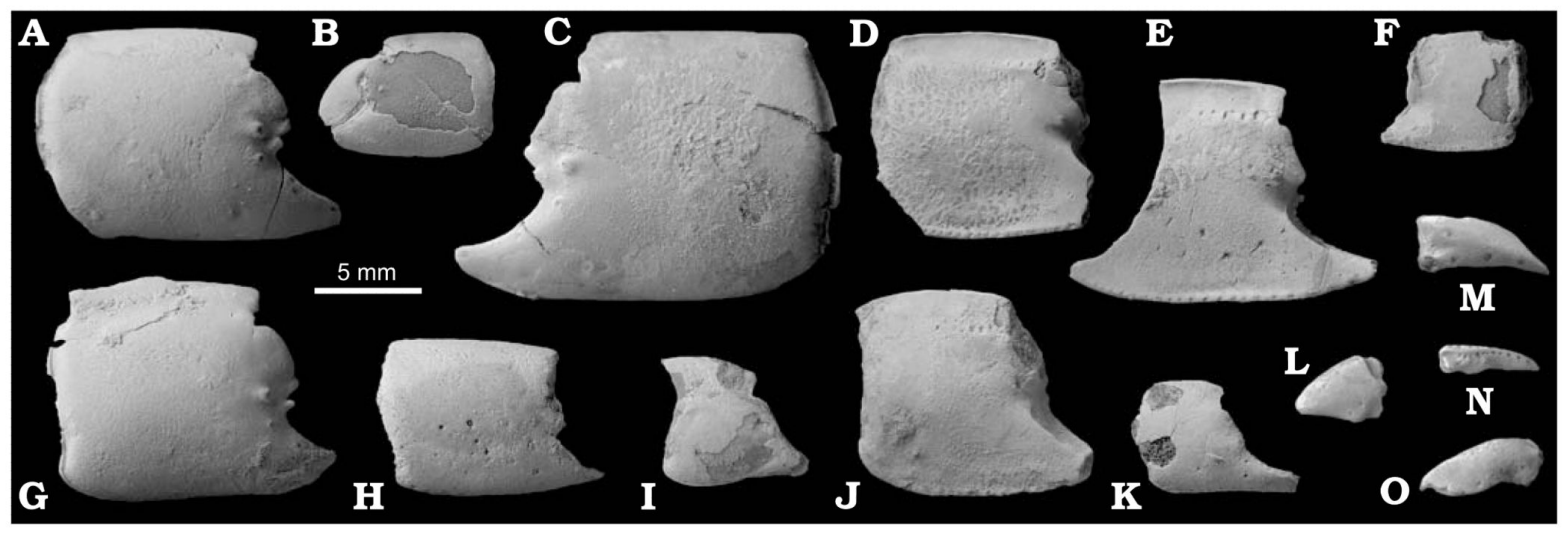
Fossorial shrimp *Lepidophthalmus crateriferus* (Lörenthey in Lörenthey and Beurlen, 1929) comb. nov., Óbuda in Budapest, Late Kiscellian; presumed male morphotypes unless stated otherwise. **A**. Right major propodus (KGP-MH OT-007). **B**. Left major propodus articulated with dactylus of presumed female (KGP-MH OT-003). **C**. Left major propodus (KGP-MH OT-009). **D**. Left major propodus (KGP-MH OT-006). **E**. Fragmentary left major propodus (KGP-MH OT-008). **F**. Right major propodus (KGP-MH OT-010). **G**. Right major propodus (KGP-MH OT-001). **H**. Right major propodus of presumed female (KGP-MH OT-002). **I**. Right minor propodus of indeterminate sex (KGP-MH OT-011). **J**. Left major propodus of presumed female (KGP-MH OT-005). **K**. Right minor propodus of indeterminate sex (KGP-MH OT-004). **L**. Left major dactylus (KGP-MH OT-017). **M**. Right major dactylus (KGP-MH OT-013). **N.** Right minor(?) dactylus (KGP-MH OT-012). **O**. Left major dactylus (KGP-MH OT-016). All elements are depicted in lateral aspect except D–F and J which are depicted in mesial view. All specimens are figured to the same scale and were covered with ammonium chloride prior to photography. Photographs by MH.

The material of *Callianassa brevimanus* Beurlen, 1939 clearly has a proximal meral hook and a distal unarmed meral blade (Fig. 2C_2_, C_3_), which are characteristic of *Lepidophthalmus*. All other morphological aspects are consistent with with this assignment, notably, the tuberculated area at the base of the fixed finger, a propodal distal tooth and morphology of the minor chela. Some of these characters are shared with *Callianopsis*, namely tubercles at the base of the fixed finger and a propodal notch with a distal tooth. The morphology of the minor cheliped is, however, distinctly different in both taxa. One specimen of *C. brevimanus* (HNHM M.59.4720; Fig. 2D) that also possesses a minor chela clearly points to the assignment of the species to *Lepidophthalmus*. Similarly, the material of *C. craterifera* consisting of isolated propodi shows above mentioned characters known in both *Callianopsis* and *Lepidophthalmus*; several specimens, however, exhibit features which are consistent with their identification as minor chelae of *Lepidophthalmus* (Fig. 3I, K).

#### Stratigraphic and geographic range

Oligocene–Holocene. Until now the only supposed fossil occurrence of the genus has been *L. jamaicense*? from the Upper Pleistocene of Jamaica reported by Collins et al. (2009). This occurrence, however, should be questioned, as only a single left propodus was found. On its basis, therefore, the determination is obscure. Collins et al. (2009) argued for its similarity to *L. jamaicense* figured by Felder and Manning (1997: fig. 3). In fact, at least two more taxa, *Sergio mericeae* Manning and Felder, 1995 and *S. sulfureus* Lemaitre and Felder, 1996, are also very similar (Manning and Felder 1995: fig. 1b; Lemaitre and Felder 1996: fig. 3a; respectively). Moreover, the material identified as?*Neocallichirus* sp. and *Neocallichirus peraensis* from the same locality seems to fall within the morphological variation of the above mentioned *Sergio* species. As a consequence, all the callianassid material reported by Collins et al. (2009) seems to represent a single taxon seemingly conspecific with one of the *Sergio* species.

*Lepidophthalmus crateriferus* comb. nov. is considered to be the first reported and oldest fossil occurrence of its genus. The genus today is widespread in the West Atlantic and In-do-West Pacific; one species, *L. turneranus* (White, 1861) is known also from the East Atlantic (Sakai 2005). The material described here may suggest the Tethyan origin of the genus; however, without any other evidence we are hesitant to draw a firm conclusion.

### *Lepidophthalmus crateriferus* (Lörenthey in Lörenthey and Beurlen, 1929) comb. nov

Figs. 2, 3.

1929 *Calianassa* [sic] *craterifera* sp. nov.; Lörenthey in Lörenthey and Beurlen 1929: 61, pl. 2: 12.
1929 *Callianassa craterifera* Lörenthey in Lörenthey and Beurlen 1929; Glaessner 1929: 79.
1939 *Callianassa brevimanus* sp. nov.; Beurlen 1939: 142, text-fig. 2, pl. 7: 5, 6.
1939 *Callianassa craterifera* Lörenthey in Lörenthey and Beurlen, 1929; Beurlen 1939: 143.
2010 *Callianassa brevimanus* Beurlen, 1939; Schweitzer et al. 2010: 34.
2010 *Callianassa craterifera* Lörenthey in Lörenthey and Beurlen, 1929; Schweitzer et al. 2010: 34.

#### Type material

Repeated search for the type material of *Callianassa craterifera* Lörenthey in Lörenthey and Beurlen, 1929, which was supposed to be deposited in the Hungarian Geological Institute in Budapest, was not successful, and thus we consider it lost. Beurlen (1939) did not designate a holotype for *Callianassa brevimanus*, so all his specimens are syntypes and we hereby designate HNHM M.59.4684a (a near complete major cheliped; Fig. 2C_1_) as the lectotype. The remaining specimens are paralectotypes (HNHM M.59.4683, M.59.4684b, M.59.4685, and M.59.4690). We hereby also select the lectotype of *C. brevimanus* to be the simultaneous neotype of *Callianassa craterifera* Lörenthey in Lörenthey and Beurlen, 1929. This action makes the *C. brevimanus* as an objective junior synonym of *C. craterifera*.

#### Type horizon

Upper Kiscellian (lowermost Chattian), Kiscell Clay Formation.

#### Type locality

Újlak brickyard at Óbuda, Budapest (site no longer available for study).

#### Other material

A single specimen showing a near-complete major cheliped together with a partially preserved minor one (HNHM M.59.4720); numerous cheliped fragments consisting of isolated propodi (HNHM INV 2012.01 [collective number], KGP-MH OT-001–011), and dactyli (KGP-MH OT-012–017); and several uncatalogued fragmentary specimens deposited in the Hungarian Geological and Geophysical Institute, Budapest.

#### Emended diagnosis

Strongly heterochelous callianassid shrimp; major cheliped merus ovoid and keeled laterally, lower margin of merus with small hook proximally and rounded blade distally; carpus shorter than high, subrectangular with oblique lower margin; propodus broad, with keeled lower and upper margins, length of fixed finger approximately one-half length of palm; palm square, with several rounded tubercles laterally and with row of elongated setal pits in the upper part of mesial surface; supposed male morphotype propodus with distally directed tooth, tooth usually undercut by broad notch at base of fixed finger, fixed finger triangular with rounded tip; dactylus high and robust, occlusal margin with large molariform tooth; supposed female morphotype propodus without tooth and notch, smoothly passing to fixed finger, lower margin of propodus convex at articulation with fixed finger.

#### Description

Major cheliped of presumed male massive. Merus ovoid, length about two times height, upper margin distinctly convex, lower margin with small sharp hook proximally and rounded blade distally (Fig. 2A, C), lateral surface with keel at midline or closer to the upper margin. Carpus distinctly shorter than high, subrectangular with straight upper and oblique lower margin, both terminated distally in angular corners (Fig. 2A, C_1_, D). Propodus broad, heavy, length of fixed finger subequal to or slightly exceeding one-half length of palm, articulation with carpus occupies the entire proximal margin. Palm square, slightly longer than high, lateral surface strongly convex with several rounded tubercles positioned at base of articulation with dactylus (e.g., Figs. 2B, 3A, C, G), tubercles with setal pits resembling small craters, mesial surface flat, in upper part with row of up to ten large setal pits positioned parallel to each other (Figs. 2E, 3D, E, J); upper and lower margins of propodus distinctly keeled, keel on upper margin bent mesially in its proximal half, keel on lower margin bent gently mesially in its entire length; lower margin with setal pits arranged in regular distances; proximal margin straight; distal margin with subtriangular, distally directed tooth, tooth usually undercut by broad notch at base of fixed finger. Fixed finger triangular with rounded tip, tip sometimes bent gently upward, with well defined lateral and mesial margins, lateral one with serrated keel (Fig. 2B). Dactylus high and robust, upper margin strongly convex, occlusal margin with large molariform tooth, sometimes subdivided, tip sharp and bent downward, lateral surface of dactylus with large setal pits (e.g., Fig. 2A, C_1_).

Major cheliped of presumed female very similar to presumed male in virtually all aspects. Differences concern mainly the shape of propodus: distal margin of propodus without tooth and notch, smoothly passing into fixed finger (Fig. 3B, H); lateral surface of propodus less armed. Lower margin convex at articulation with fixed finger.

Propodus of presumed minor cheliped higher than long, upper margin convex, distal margin smoothly passing to fixed finger; narrow fixed finger as long or slightly longer than palm (Fig. 3I, K); dactylus long, with distinct setal pits.

Dorsal carapace, abdomen and other appendages unknown.

#### Discussion

Lörenthey in Lörenthey and Beurlen (1929) described *Calianassa* [sic] *craterifera* on the basis of seven well preserved isolated propodi from the Upper Oligocene brickyard in Eger (Bondor 1964; Kenawy and Nyírö 1967). Later, Beurlen (1939) described *Callianassa brevimanus* on the basis of several well preserved specimens from the Kiscell Clay. Unfortunately, he did not recognize common features between his species and *C. craterifera*, although he mentioned the latter taxon in his work. Both taxa share a general shape of the propodus, similar tuberculation on the lateral surface of the propodus at the articulation with dactylus, and also distinctive setal pits on the inner surface of propodus just below its upper margin (presence of similar setal pits have been figured also in *Lepidophthalmus turneranus* [de Man 1928: fig. 21c]). These pits which are present on the mesial surface of the propodus are not mentioned by Beurlen (1939). In most samples of *C. brevimanus* the specimens are preserved embedded in matrix usually with the lateral surface exposed, so the setal pits are therefore usually obscured by sediment. Only in one specimen, which is preserved as an imprint of the mesial surface, are these setal pits visible, and even then only when it was covered with ammonium chloride (Fig. 2E). Beurlen (1939: pl. 7: 5) figured the same specimen, but the pits are, however, not discernible. In *C. craterifera* the pits have been sufficiently described and figured by Lörenthey in Lörenthey and Beurlen (1929: 62, pl. 2: 12). As a result, on the basis of morphological similarities together with roughly the same age of both taxa, *C. brevimanus* and *C. craterifera* are considered synonymous, and reassigned to *Lepidophthalmus* as discussed above.

*Lepidophthalmus crateriferus* comb. nov. differs from all extant congeners. Many extant *Lepidophthalmus* species possess a proximally situated U-shaped notch on the upper margin of the merus which *L. crateriferus* comb. nov. lacks. The distal blade on the lower margin of merus is not denticulated as it is in many extant taxa. *Lepidophthalmus crateriferus* comb. nov. possesses a rather short carpus and a massive strongly vaulted propodus, and in this respect, it is closest to *L. rosae* (compare Sakai 2005: fig. 31A–C). *Lepidophthalmus crateriferus* comb. nov. has a deep dactylus with a single large molariform tooth (or keel) on the occlusal margin; such an armature is considered unique among *Lepidophthalmus* species.

#### Stratigraphic and geographic range

The species is so far known only from the Late Oligocene of Hungary.

### Family Ctenochelidae Manning and Felder, 1991

#### Discussion

The family Ctenochelidae was erected by Manning and Felder (1991) to accommodate several genera previously classified within the family Callianassidae. De Grave et al. (2009) listed seven ctenochelid genera in four independent subfamilies, Callianopsinae Manning and Felder, 1991, Ctenochelinae Manning and Felder, 1991, Gourretiinae Sakai, 1999a and Pseudogourretiinae Sakai, 2005. Sakai (2011) elevated the subfamilies to familial status, thus leaving Ctenochelidae as containing *Ctenocheles* only. Recently, *Ctenocheloides attenboroughi* Anker, 2010, a new ctenochelid genus and species, has been described from very shallow marine environments of Madagascar.

### Genus *Ctenocheles* Kishinouye, 1926

#### Type species

*Ctenocheles balssi* Kishinouye, 1926, by monotypy; Ohsu near Kashiwasaki, Niigata Prefecture, Japan.

#### Species included

See Table 2.

#### Emended diagnosis

Rostral carina and rostral spine present; dorsal surface of eye flattened; third maxilliped with or without exopod, distal margin of merus usually with spine; chelipeds unequal, and dissimilar; major cheliped carpus small, cup shaped; major cheliped merus with or without hook; palm of major cheliped bulbous, longer than high, narrowing distally; fingers elongate and pectinate; fixed finger straight or arcuate; occlusal surface of fixed finger with long, needle-like teeth, teeth of variable size, tips curving proximally. Palm of minor cheliped rectangular; fixed finger long, narrow, straight; uropodal exopod with lateral incision (emended from Manning and Felder 1991: 784).

#### Discussion

*Ctenocheles* is a poorly known genus. Although six nominate species have been described from extant environments (Table 2), virtually all of them are based on a handful of specimens (Kishinouye 1926; Ward 1945; Powell 1949; Rodrigues 1978; Rabalais 1979; Matsuzawa and Hayashi 1997; Sakai 1999a). The best known taxon seems to be *C. balssi* (the type species), in which a statistically robust amount (40) of detached major chelipeds were also examined (Matsuzawa and Hayashi 1997). Complete animals are rarely found whereas detached chelipeds usually are collected (Balss 1914; Holthuis 1967; Crosnier 1969). Similarly the fossil record of the genus consists almost exclusively of its chelae (Schweitzer and Feldmann 2001). *Ctenocheles secretanae* Schweitzer and Feldmann, 2002 and *C. rupeliensis* (Beurlen, 1939), known from near-complete animals are notable exceptions.

The typical shape of the major propodus and dactylus, i.e., bulbous palm with long pectinate fingers, usually allow specimens to be immediately assigned to the genus, and therefore the genus is easily recognizable; the minor chelipeds are less significant. Minor chelipeds may be misinterpreted, and this has happened previously in *Ctenocheles rupeliensis*, as documented below. No sexual dimorphism in major cheliped morphology of *Ctenocheles* is known (Matsuzawa and Hayashi 1997).

*Ctenocheloides* Anker, 2010 has a similarly shaped major cheliped, but its fingers are distinctly shorter than in *Ctenocheles*. Moreover, *Ctenocheloides* has weakly unequal and asymmetrical chelipeds, whereas *Ctenocheles* is strongly heterochelous.

Tshudy and Sorhannus (2000) studied evolutionary trends in the occurrence of pectinate chelipeds in decapod crustaceans. They postulated convergence in four lineages. In the current classification (De Grave et al. 2009) two of them are nephropid lobsters (Astacidea), one is a palaeopentachelid (Polychelida) and the other is *Ctenocheles* (Axiidea). Other examples of convergent development of pectinate chelae can be found in astacidean families Stenochiridae (*Stenochirus* Oppel, 1861) (e.g., Schweigert et al. 2006) and Erymidae (*Lissocardia* Von Meyer, 1851) (e.g., Garassino et al. 1999) and brachyuran families Leucosiidae Samouelle, 1819 and Iphiculidae Alcock, 1896.

Discussion on the fossil record, palaeobiogeography and palaeoecology of *Ctenocheles* was provided by Förster and Mundlos (1982), Feldmann et al. (1995), Tshudy and Sorhannus (2000), and Schweitzer and Feldmann (2001, 2002).

#### Stratigraphic and geographic range

Cenomanian to Holocene. Two species are known from the Late Cretaceous, *C. madagascariensis* Secrétan, 1964 (recently re-examined by Charbonnier et al. 2012) and *C. inaequidens* (Pelseneer, 1886) from Madagascar and the Netherlands, respectively. The genus has been widely reported from the Cenozoic from all over the world. Today, there are 6 named and a few un-named species known worldwide except from the eastern Pacific (Sakai 1999a, b, 2005, 2011) (Table 2). Burukovsky (2005) described *Thaumastochelopsis plantei* Burukovsky, 2005 on the basis of a single specimen from the continental shelf of Madagascar. However, the animal apparently does not represent a lobster, but an axiidean shrimp, most probably a member of *Ctenocheles* (Chan 2010: 156).

### *Ctenocheles rupeliensis* (Beurlen, 1939)

**Fig. 4:**
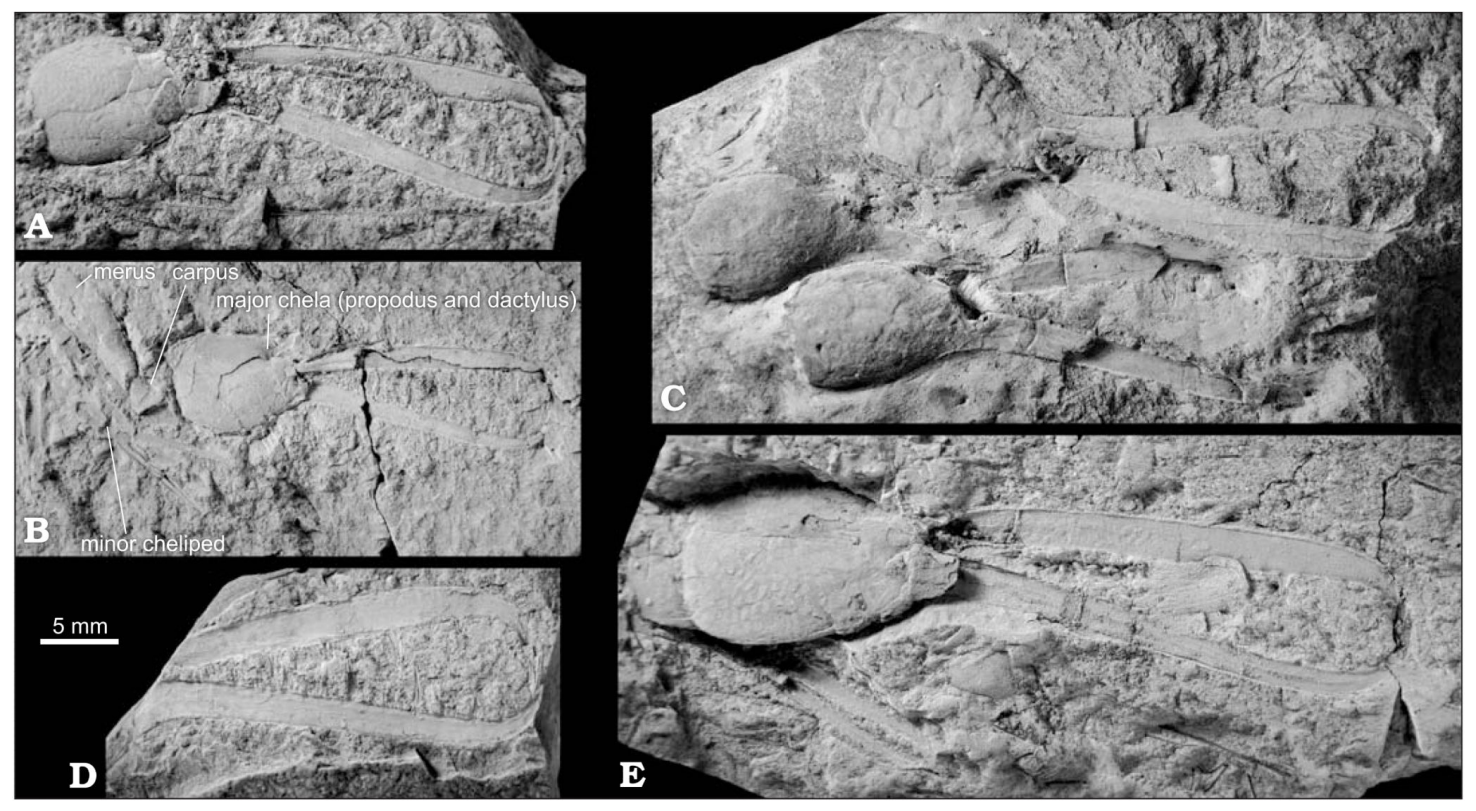
Fossorial shrimp *Ctenocheles rupeliensis* (Beurlen, 1939), Óbuda in Budapest, Late Kiscellian. **A**. Right major cheliped (HNHM M.66.961). **B**. Specimen with both chelipeds preserved, lectotype selected herein (HNHM M.59.4696a). **C**. Accumulation of three isolated major chelae (HNHM M.59.4703). **D**. Pectinate fingers of major chela (HNHM M.59.4696). **E**. Specimen with both chelipeds preserved (HNHM M.59.4704). Note elongated shape of the propodus. All specimens except HNHM M.59.4696a are paralectotypes selected herein. All specimens are figured to the same scale and were covered with ammonium chloride prior to photography. Photographs by MH.

**Fig. 5:**
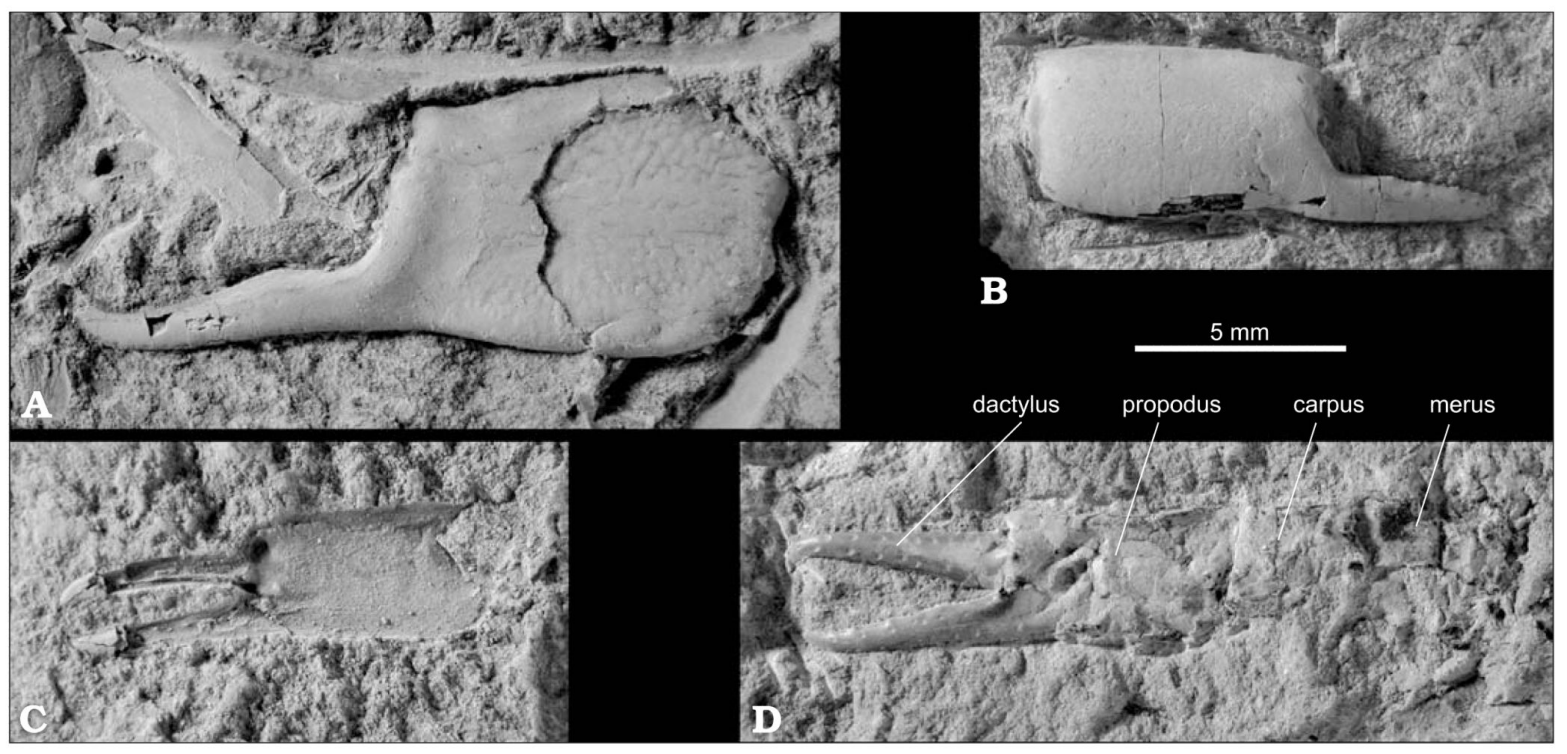
Minor chelae of fossorial shrimp *Ctenocheles rupeliensis* (Beurlen, 1939), Óbuda in Budapest, Late Kiscellian. **A**. Left minor propodus (HNHM M.59.4700). **B**. Right minor propodus (HNHM M.59.4869). **C**. Minor propodus articulated with dactylus (HNHM M.59.4691). **D**. Articulated left minor chela (HNHM M.59.4682). All specimens are paralectotypes selected herein. All specimens are figured to the same scale and were covered with ammonium chloride (except D) prior to photography. Photographs by MH.

**Fig. 6:**
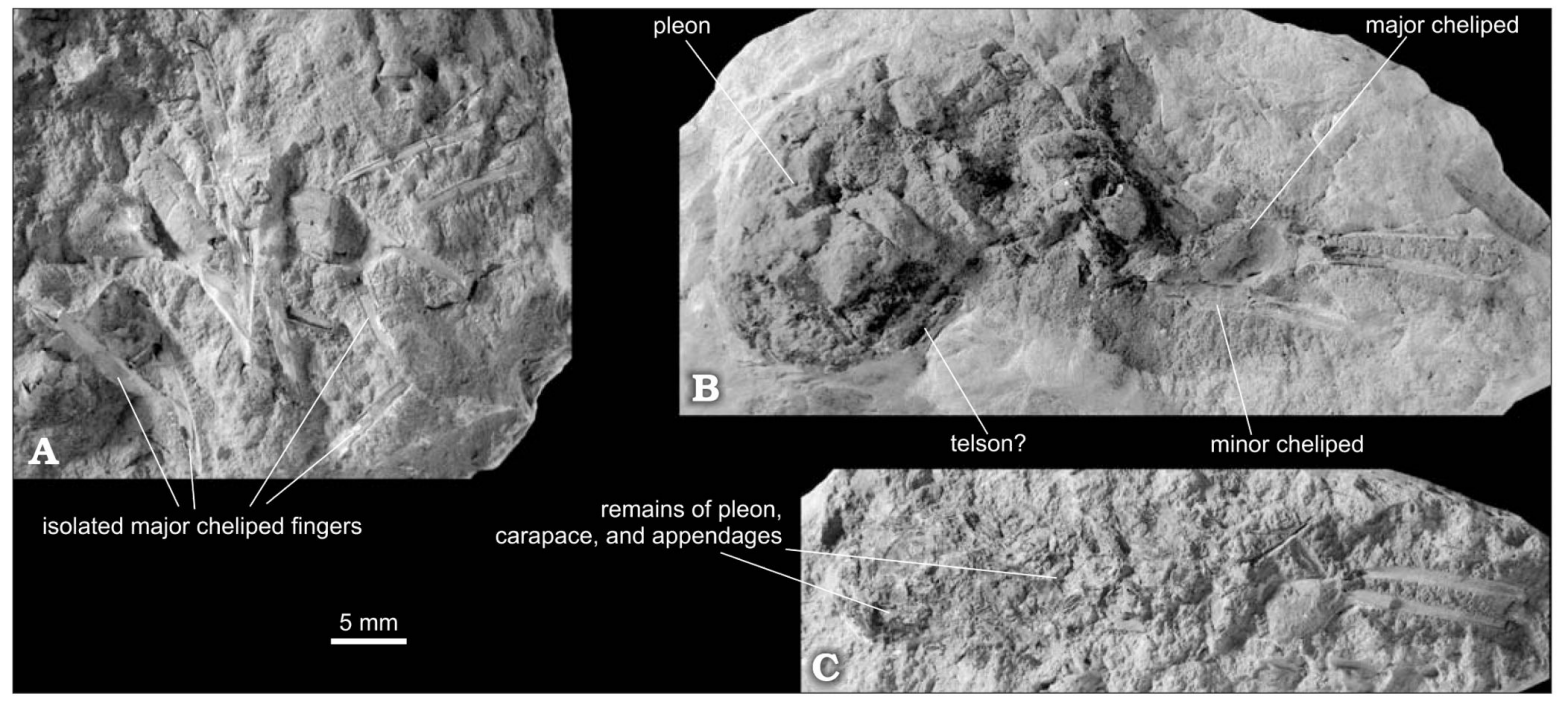
Fossorial shrimp *Ctenocheles rupeliensis* (Beurlen, 1939), Óbuda in Budapest, Late Kiscellian. **A**. Mass accumulation of isolated major cheliped fingers (HNHM M.59.4706). **B**, **C**. Near complete specimens with preserved carapaces, pleons and appendages. **B**. HNHM M.59.4709. **C**. HNHM M.59.4694b. All specimens are paralectotypes selected herein. All specimens are figured to the same scale. Photographs by MH.

Figs. 4A–E, 5A–D, 6A–C.

1939 *Thaumastocheles rupeliensis* sp. nov.; Beurlen 1939: 137, text-fig. 1, pl. 7: 1, 2.
1939 *Callianassa nuda* sp. nov.; Beurlen 1939: 144, text-fig. 3, pl. 7: 3, 4.
1941 *Thaumastocheles rupeliensis* Beurlen, 1939; Mertin 1941: 179, 185, fig. 10q.
1957 *Thaumastocheles rupeliensis* Beurlen, 1939; Imaizumi 1957: 303.
1996 *Ctenocheles* cf. *rupeliensis* (Beurlen, 1939); Polkowsky 1996: 54.
2000 *Ctenocheles rupeliensis* (Beurlen, 1939); Tshudy and Sorhannus 2000: 481, 484.
2002 *Ctenocheles rupeliensis* (Beurlen, 1939); Moths and Montag 2002: 6, pl. 5: 2-7.
2003 *Ctenocheles* sp.; Mikuž 2003: 90, pl. 1: 1–5.
2004 *Ctenocheles chattiensis* sp. nov.; Polkowsky 2004: 27, pl. 4: 17-27.
2010 *Callianassa nuda* Beurlen, 1939; Schweitzer et al. 2010: 36.
2010 *Ctenocheles chattiensis* Polkowsky, 2004; Schweitzer et al. 2010: 40.
2010 *Ctenocheles rupeliensis* (Beurlen, 1939); Schweitzer et al. 2010: 40.

#### Type material

Lectotype selected herein: HNHM M.59.4694a, paralectotypes: HNHM M.59.4682, M.59.4686, M.59.4689, M.59.4691–4693, M.59.4694b, M.59.4696–4697, M.59.4700–4701, M.59.4703–709, M.59.4712, M.66.961.

#### Type horizon

Upper Kiscellian (lowermost Chattian), Kiscell Clay Formation.

#### Type locality

Újlak brickyard at Óbuda, Budapest (non existent anymore).

#### Other material

Single fragmented major propodus (FI.1339) and numerous uncatalogued cheliped fragments deposited in the Hungarian Geological and Geophysical Institute, Budapest.

#### Emended diagnosis

Major cheliped merus long and slender, unarmed, narrowing in both ends; fixed finger at angle of about 20–40° to the long axis of palm fingers about 1.5–2.5 length of palm; both fingers armed with long, needle-like teeth with three sizes, between two large teeth there are one to five small and medium teeth alternating with each other; tips of fingers strongly curved proximally forming large teeth crossing each other and exceeding at least twice the length of the large teeth on the occlusal surface.

#### Description

Chelipeds distinctly unequal in size and dissimilar in shape. In major cheliped, merus slender, unarmed, narrowing in both ends, approximately as long as carpus and palm together (Fig. 4B); carpus short, higher than long, and cup-shaped (Fig. 4B); palm bulbous, rounded or slightly elongate, longer than high, narrowing distally; fingers slender and elongate, about 1.5–2.5 times as long as palm, fixed finger at angle of about 20–40° to the long axis of palm, occlusal surface of both fingers armed with long, needle-like teeth with three sizes (Fig. 4), between two large teeth there are one to five small and medium teeth alternating with each other; tips of fingers strongly curved proximally forming large teeth crossing each other and exceeding at least twice the length of large teeth on occlusal surface.

Minor cheliped slender, less massive than larger cheliped (Fig. 5); carpus higher than long, with rounded proximo-lower margin (Fig. 5D); palm rectangular, longer than high, only slightly tapering distally; fixed finger long, narrow and straight, approximately as long as palm, occlusal margin of both fingers armed with a row of denticles, occlusal margin of fixed finger usually with proximal concavity (e.g., Fig. 5A).

Dorsal carapace, pleon, and other appendages insufficiently preserved.

#### Intraspecific variation

Studied material shows variability in the shape of the palm of both major and minor chelae. The major cheliped palm can be nearly globular (Fig. 4A, B) or slightly elongated (Fig. 4E), and usually it is longer than high. The minor cheliped palm is usually distinctly longer than high with near-parallel upper and lower margins; in some specimens, though, the palm is shorter with upper and lower margins that are seemingly convex (Fig. 5A), thus resembling the bulbous nature of the major palm. The length of the fingers is also rather variable. Most specimens have fingers that are approximately two times longer than palm; however, some are distinctly longer, up to 2.5 times longer than palm (similar to extant *C. balssi* Kishinouye, 1926 and *C. leviceps* Rabalais, 1979), and one specimen (HNHM M.59.4705) has a ratio of only 1.5 (similar to extant *C. collini* Ward, 1945). The occlusal surfaces of both major cheliped fingers are usually are armed with three teeth sizes; the pattern of alternating small and medium teeth between two large ones is variable depending on the distance of teeth from the proximal end; in the middle portion of fingers the teeth are usually more numerous (cf. Glaessner 1960). No constant formula can be given except that there are between 1 and 5 (usually 2–3) smaller teeth between two large ones. Similarly the dentition in the minor cheliped is variable; it may consist of two alternating sizes of teeth, or of teeth of uniform size.

#### Discussion

*Ctenocheles rupeliensis* was described by Beurlen (1939) as a member of *Thaumastocheles* (Astacidea: Nephropidae). It should be noted that *Ctenocheles balssi*, the type species of *Ctenocheles*, was described on the basis of material ascribed by Balss (1914) to ?*Pentacheles* nov. sp. Beurlen (1939) drew attention to the striking resemblance of his *Thaumastocheles rupeliensis* to the specimen reported by Balss (1914); thus, he clearly recognized the identity of the material, although he did not mention Kishinouye’s work. Later, the species was formally recognized (Glaessner 1947) to be a member of *Ctenocheles*.

Beurlen (1939) described the pectinate fingers and propodus of the major cheliped of this species and paid no attention to other preserved parts of the animal. Tshudy and Sorhannus (2000) mentioned that only a few claws of *C. rupeliensis* had been described. The original material, however, is far richer. In two studied specimens virtually the entire animal is preserved (Fig. 6B, C). Unfortunately, details of soft-part morphology are obscured because of insufficient preservation.

Beurlen (1939) described *Callianassa nuda* on the basis of several mostly isolated cheliped fragments showing the palm as distinctly longer than high and with relatively long fingers. The material can be attributed to the minor chelae of *Ctenocheles* (Fig. 5); they are, thus, considered conspecific with *C. rupeliensis*.

Differentiation between fossil species of *Ctenocheles* was discussed by several authors. Collins and Jakobsen (2003) distinguished *Ctenocheles anderseni* Collins and Jakobsen, 2003 from other northern European congeners on the basis of differences in the arrangement of the denticles lining the occlusal margin of dactylus. Feldmann et al. (2010: 341) argued that, “the outline of the manus; the height of the fixed finger; the longitudinal profile of the fixed finger, whether straight or curved; the form of the denticles on the occlusal surface; and form of the proximal part of the fixed finger are characters diagnostic of species within the genus”. Unfortunately, the intraspecific variation in finger dentition is poorly known. For instance, Glaessner (1960) reported in *Ctenocheles* cf. *maorianus* from the Late Pleistocene of New Zealand three to four small teeth between the large ones in the middle portion of the fingers of the major chela but up to six small teeth in the intervals on larger fingers. No tooth formula has been stated in descriptions of extant taxa and on the basis of isolated fingers the taxa probably are difficult, if not impossible, to differentiate from each other. For instance, tooth arrangements in *C. balssi* and *C. leviceps* according to published figures (Sakai 1999a: fig. 2b, and Rabalais 1979: 15–17, respectively) are indistinguishable.

Matsuzawa and Hayashi (1997) provided a key for extant *Ctenocheles* species. Among other characters they considered the morphology of the major cheliped ischium and merus, as well as the ratio between the length of the palm and fingers, as characters on which basis nominate taxa can be distinguished. Large numbers of entire chelae preserved in *Ctenocheles rupeliensis* allows for an estimation of intraspecific variation in this species. Although many propodi of studied material are partially compressed, they clearly have rather variable outlines, from almost rounded to more elongate. Interestingly, specimens exhibit variable ratios between the length of the palm and fingers (see above). Similarly, there is rather great variability in the arrangement of teeth on occlusal margins of fingers.

Feldmann et al. (2010) distinguished *C. notialis* from the Miocene–Pliocene of Chile also on the basis of the angle of the fixed finger. In their diagnosis of *C. notialis* they noted the angle of the fixed finger to the long axis of the palm to be 35°. One of the figured specimens (Feldmann et al. 2010: fig. 3A), however, clearly shows an angle of about 50°. Thus, the material exhibits angle values which overlap with other *Ctenocheles* species. For instance the material of *C. rupeliensis* shows a range of an angle values 20–40°.

As a result we conclude that the shape of the propodus, the ratio between the length of the palm and fingers, the dentition of fingers, and the angle of the fixed finger are intraspecifically variable characters which are uninformative on the species level if not treated in combination with other characters. The problem seems to be even broader as the comparison of extant *Ctenocheles* species clearly shows major differences in the nature of the major cheliped ischium and merus. When summarizing these characters one can distinguish three cheliped morphotypes present in extant *Ctenocheles*: (i) ischium and merus elongate, slender and completely unarmed (*C. balssi*; *C. leviceps*; *Ctenocheles* sp. A sensu Holthuis, 1967; *Ctenocheles* sp. B sensu Holthuis, 1967); (ii) ischium serrated; merus ovoid with distinctly convex upper margin, unarmed (*C. collini, C. maorianus*); (iii) ischium with spines on lower margin; merus elongate with single median tooth on lower margin (*C. holthuisi*). *Ctenocheles serrifrons* is not included in this summary, as the major cheliped is unknown in this species (Le Loeuff and Intès 1974). If one follows Manning and Felder (1991) in considering the merus as of taxonomic importance, then one would interpret these three morphological groups as separate genera.

*Ctenocheles rupeliensis* clearly can be assigned to the first morphological group as it possesses an elongate and completely unarmed merus (Fig. 4B). As this group is defined mostly by *C. balssi*, the type species of *Ctenocheles*, we are hesitant to deal with the generic assignment of the rest of morphotypes as listed above without proper examination of their soft part morphology.

Mikuž (2003) reported cheliped fragments ascribed to *Ctenocheles* sp. from the Oligocene of Slovenia. Considering the relative geographical proximity of the Hungarian Kiscell Clay localities these might represent *C. rupeliensis*. The material itself is, however, too fragmentary to judge with confidence.

Polkowsky (2004) erected a new species, *Ctenocheles chattiensis*, from the Late Oligocene of Northern Germany. Although this material is slightly younger than *C. rupeliensis*, we consider it to be conspecific, although its preservation does not allow for much comparison. In fact it is questionable whether the material can form a basis for a new taxon. Supposed morphological differences as stated by Polkowsky (2004), namely the shape of lower and proximal margins of the palm of both major and minor chelipeds, are variable features. Polkowsky (2004) stressed the presence of two rows of setal pits along the fingers of the major cheliped which are actually present in all callianassoid shrimps and can not be considered as characters of taxonomic importance at the species level. Interestingly, Moths and Montag (2002) reported the presence of *C. rupeliensis* from the type locality (Kobrow) of *C. chattiensis* as stated by Polkowsky (2004). The material from a different locality (Malliss) reported by Moths and Montag (2002) exhibits more of the preserved characters than the material of Polkowsky (2004) does. As a result, *C. chattiensis* is considered herein a junior synonym of *C. rupeliensis*.

There are several *Ctenocheles* species described from the Eocene and Oligocene of Italy (Table 2). Direct comparison with *C. rupeliensis* is difficult, as all of them are described on the basis of propodi and dactyli only (which are subjects of intraspecific variation), and no merus or ischium has been described so far.

#### Stratigraphic and geographic range

The species is known from the Oligocene of Hungary and Northern Germany.

## Discussion

### Taphonomy

Some of the nautiloid shells of the Kiscell Clay were buried in a perpendicular position, which implies extremely calm, almost motionless bottom water (Báldi 1986). This conclusion is in accordance with the state of preservation observed in the ghost shrimps. Several specimens of *Ctenocheles rupeliensis* retain the carapace and pleon, which are not usually present in the fossil record. Moreover, virtually all chelipeds are preserved articulated and no isolated finger fragments have been recovered. In several cases both chelae are preserved very close to each other. Similarly, in *Lepidophthalmus crateriferus* comb. nov. several specimens retain near-complete chelipeds and in one case a minor chela is preserved close to the major one. All these observations suggest a rather rapid burial without subsequent physical or biological disturbance; thus it is autochtonous or parautochtonous. Cuticular surfaces of callianassoid shrimps are fragile and soon after death of an animal the body is usually disintegrated (Bishop and Williams 2005). As a consequence no scavenging and/or subsequent physical disturbance can be inferred for the depositional conditions in which the studied ghost shrimps were preserved.

### Palaeoecology and palaeobathymetry of the Kiscell Clay

The planktonic foraminifers of the Kiscell Clay recollect colder northern-European foraminiferan associations rather than the warm-water Mediterranean faunas (Báldi 1983; Horváth 1998). On the other hand the living relatives of the Kiscell Clay fishes live in subtropical climates.

A normal marine environment is indicated for the Kiscell Clay by the relatively diverse fossil associations. Earlier, this formation was thought to be deposited in shallow water environment (e.g., Sztrákos 1974); however, on the basis of the mollusc association Báldi (1986) argued for a shallow bathyal environment. The deep-water fauna of the Kiscell Clay consists of mollusc genera *Aporrhais* Costa, 1778, *Tibia* Röding, 1798, *Galeodea* Link, 1807, *Athleta* Conrad, 1853, *Turricula* Schumacher, 1817, *Nuculana* Link, 1807, *Cuspidaria* Nardo, 1840, *Pseudamussium* Mörch, 1853, and *Limopsis* Sassi, 1827. The trophic structure of the mollusc fauna implies disphotic depths, as suspension filters, carnivores and deposit feeders build up the assemblage while the herbivores are absent (Báldi 1986). This conclusion is in concordance with the dominance of *Ctenocheles rupeliensis* in the decapod assemblage, as individuals of *Ctenocheles* are typically blind.

Báldi (1986) correlated the Kiscellian fauna (dominated by *Cultellus budensis* Báldi, 1973 and *Propeamussium* de Gregorio, 1884) with the *Propeamussium simile*–*Abra longicollis* community inhabiting the Adriatic Seaat a depth of 150–400 m depth.

A deep-water environment for the Kiscell Clay is also indicated also by other faunal elements. The foraminiferan assemblages suggest a deeper water origin on the basis of comparison to extant forms with known ecological requirements, the plankton/benthos ratio, and the ratio of hyaline shelled and agglutinated forms (Horváth 1998, 2002). These data suggest a depth of several hundred meters; the minimum depositional depth of the upper part of the Kiscell Clay might have been 200 m and the maximum depth can be estimated at 600–1000 m (middle bathyal zone) (Horváth 1998). The depth of the Kiscell Sea and the oxygen level of the bottom water were recently studied by Sóron (2008) at Felsöpetény (65 km NE of Budapest). On the basis of quantitative and qualitative analysis of the agglutinated foraminifers the lower part of the Kiscell Clay was deposited in the upper bathyal zone, where the bottom water was dysoxic. Concerning the ecological requirements of *Lepidophthalmus*, it is able to tolerate prolonged hypoxia (Felder 1979; Felder and Manning 1998).

The ostracod fauna of the Kiscell Clay is suggestive of normal saline, mainly bathyal environment (Monostori 2008). Cirripeds are represented by the bathyal genus *Scalpellum*, which most probably cemented to swimming organisms and then accumulated in deep-water sediments (Szörényi 1934). A typical deeper-water coral, the fan-shaped *Flabellum* Lesson, 1831 was mentioned from the Kiscell Clay by Hegedüs (1962). The quiet, deep-water environment of the Kiscell Clay is also confirmed by accumulation of several articulated thin shelled echinoid tests. The brachiopod *Terebratulina* d’Orbigny, 1847 is also a member of deeper-water assemblages (Logan 1979). The Kiscell Clay from NE Hungary has provided an association of deep-water fishes, quantitatively very rich in otoliths of mesopelagic fishes (Nolf and Brzobohatý 1994).

According to Báldi (1986) the rate of sedimentation can be roughly 400-500 m/Ma in the Kiscell Clay. On the basis of different arguments, he proposed a sedimentary depth between 200 and 1000 m for the Kiscell Clay.

Concerning the bathymetry, the decapod association generally corroborates the results dicussed above, although if it were solely based on decapods, palaeoecological interpretation would be difficult. It is true that *Ctenocheles* today is generally considered as inhabitant of rather deep-water habitats, but its bathymetric distribution is nevertheless quite broad, ranging from 10 to approximately 800 m (Balss 1914; Holthuis 1967; Sakai 2011). Interestingly most *Ctenocheles* fossils are known from the inner continental shelf, although this may be explained by both ecological displacement towards the Recent or as a preservational bias against ancient slope and rise dwellers (Tshudy and Sorhannus 2000). On the other hand *Lepidophthalmus* is today known exclusively from shallow-water environments. Moreover, it is able to tolerate even freshwater environments (e.g., Dworschak 2007). Generally it is concentrated in intertidal and shallow subtidal substrates ranging from sandy mud to organic silty sand. Felder and Lovett (1989) characterized *Lepidophthalmus louisianensis* Schmitt, 1935 as adapted to oligohaline habitats of coastal marshes, tidal channels and estuarine embayments. Members of the genus *Lepidophthalmus* have been reported to migrate periodically up the rivers, e.g., *L. turneranus* in West Africa (Vanhöffen 1911; Monod 1927). It is rather surprising to find *Lepidophthalmus* in a deep water habitat. The brachyuran genus *Lyreidus* de Haan, 1841 (present in the Kiscell Clay with *L. hungaricus* Beurlen, 1939) is today a typical inhabitant of offshore habitats (Powell 1949; Dell 1963), although it occurs also in shallow inshore waters at diveable depths (McLay 1988; Takeda and Webber 2006). Indeed, in the fossil record it has been reported from shallow-water environments (e.g., Feldmann and Wilson 1988). Thus, the composition of the Kiscell Clay decapod assemblage itself does not necessarily imply deep-water habitat but evidence from other sources clearly identifies it as a deep-water environment.

### Shift of ecological preferences in ghost shrimps?

An on-shore-to-offshore shift in distribution, connected with shifts in ecological preferences, is known in diverse animal groups (Jablonski et al. 1984). Such a shift throughout the evolutionary history of decapod lineages is also widely known. Within one lineage, stratigraphically older taxa inhabiting shallow water environments later shift to more deep-water habitats. Ecological displacement to deeper habitats is well documented by the Cenozoic fossil record of decapod crustaceans. It has been reported in several higher taxa including polychelid lobsters (Beurlen 1931; Ahyong 2009), astacideans (Feldmann and Tshudy 1989; Tshudy and Sorhannus 2000), glypheideans (Neto de Carvalho et al. 2007) and homolodromioid brachyuran crabs (Förster et al. 1987; Feldmann and Wilson 1988; Collins 1997; Feldmann and Gaździcki 1998; Müller et al. 2000; Krobicki and Zatoń 2008). Feldmann and Wilson (1988) reported three decapod genera, *Munidopsis* Whiteaves, 1874, *Homolodromia* A. Milne Edwards, 1880, and *Lyreidus* from the Eocene shallow marine settings of Antarctica, which today are known primarily from offshore, deep-water habitats.

Possible ecological shifts have not been studied extensively in ghost shrimps, which can be attributed mainly to the poor understanding of their fossil record. Although callianassoid shrimps are one of the most common and numerous decapod fossils, their generic assignment is often obscure and consequently their evolutionary lineages are difficult to reconstruct. Both *Ctenocheles rupeliensis* and *Lepidophthalmus crateriferus* comb. nov. from the Kiscell Clay clearly were inhabitants of a deep-water environment as dicussed above. It is not surprising to find *Ctenocheles* in such an environment, but for *Lepidophthalmus* the opposite is true. From the perspective of the above discussed onshore-offshore pattern the *Lepidophthalmus* case seems to be reversed, as the representatives of this genus are known today only from very shallow water settings (see above). Two scenarios are possible: *L. crateriferus* comb. nov. may have given rise to at least some extant shallow water congeners, or it simply is a descendant of some yet unknown shallow water species whose ecological preferences shifted in accordance with discussion above. The latter scenario seems to be more probable. Without any other evidence, however, the first possibility should also be considered as possible.

## Conclusions

Taxonomic redescription of the Kiscell Clay decapod fauna focused on burrowing shrimps provides new data on the understanding of their fossil record. The variation within the material of *Ctenocheles rupeliensis* calls for the re-assessment of interspecific differences between extant and fossil species of *Ctenocheles*. The characters present on the pectinate claws (major chelipeds) are usually used for species distinction; these are, however, shown to be a subject of major intraspecific variation. The material of *Callianassa brevimanus* and *C. craterifera* allows the synonymization of respective taxa and their reassignment to *Lepidophthalmus*. The morphology of chelipeds of this genus is remarkably similar to ctenochelid *Callianopsis*. The key character proposed herein to distinguish these two genera in the fossil record is the presence of the proximal meral lobe (or blade) on the major cheliped. The studied decapod fauna is considered to come from a deep-water (bathyal) environment as inferred from other faunal elements. Finding of *Lepidophthalmus* (otherwise a typical inhabitant of a very shallow environment) in deep-water settings may be surprising; the evolutionary history of the genus is, however, virtually unknown and a shift of ecological preferences cannot be excluded in this case.

## Figures and Tables

**Table 1:** Synopsis of the taxonomy of the Kiscell Clay decapod assemblage.

Original placement	Current placement	Relevant reference
*Thaumastocheles rupeliensis* Beurlen, 1939	*Ctenocheles rupeliensis*	this paper
*Callianassa nuda* Beurlen, 1939	*Ctenocheles rupeliensis*	this paper
*Callianassa craterifera* Lörenthey in Lörenthey and Beurlen, 1929	*Lepidophthalmus crateriferus*	this paper
*Callianassa brevimanus* Beurlen, 1939	*Lepidophthalmus crateriferus*	this paper
*Lyreidus hungaricus* Beurlen, 1939	*Lyreidus hungaricus*	Beurlen (1939)
*Calappa tridentata* Beurlen, 1939	*Calappilia tridentata*	Schweitzer et al. (2010)
*Plagiolophus sulcatus* Beurlen, 1939	*Glyphithyreus sulcatus*	Karasawa and Schweitzer (2004)

**Table 2:** Synopsis of species of *Ctenocheles* known to date. Note: data on stratigraphical age and geographical distribution are supplied only for fossil occurrences.

	Age	Locality
Species with an exclusively Recent record		
*Ctenocheles balssi* Kishinouye, 1926		
*Ctenocheles collini* Ward, 1945		
*Ctenocheles holthuisi* Rodrigues, 1978		
*Ctenocheles leviceps* Rabalais, 1979		
?*Ctenocheles plantei* (Burukovsky, 2005)		
*Ctenocheles serrifrons* Le Loeuff and Intès, 1974		
*Ctenocheles* sp. A sensu Holthuis, 1967		
*Ctenocheles* sp. B sensu Holthuis, 1967		
Extant species known also from the fossil record		
*Ctenocheles maorianus* Powell, 1949	Late Pleistocene	New Zealand
Exclusively fossil species		
*Ctenocheles madagascariensis* Secrétan, 1964	Albian–Maastrichtian	Madagascar
*Ctenocheles fritschi* Hyžný, Kočová Veselská and Dvořák, 2014	Early–Middle Coniacian	Czech Republic
*Ctenocheles inaequidens* (Pelsenner, 1886)	Early Maastrichtian	The Netherlands
*Ctenocheles bakeri* (Glaessner, 1947)	Middle Paleocene (?Eocene)	Australia (Victoria)
*Ctenocheles victor* Glaessner, 1946	Late Paleocene (?Eocene)	Australia (Victoria)
*Ctenocheles cultellus* (Rathbun, 1935)	Late Paleocene/Eocene	USA (Alabama, Mississippi), ?Spain
*Ctenocheles anderseni* Collins and Jakobsen, 2003	Early Eocene	Denmark
*Ctenocheles cookei* (Rathbun, 1935)	Early Eocene	USA (Alabama)
*Ctenocheles sereaensis* Beschin, De Angeli, and Zorzin, 2009	Early Eocene	Italy
*Ctenocheles valdellae* (Fabiani, 1908)	Early–Middle Eocene/Early Oligocene	Italy
*Ctenocheles sujakui* Imaizumi, 1957	Eocene	Japan
*Ctenocheles burlesonensis* (Stenzel, 1935)	Middle Eocene	USA (Texas), ?Spain
*Ctenocheles dentatus* (Rathbun, 1935)	Middle Eocene	USA (Mississippi)
*Ctenocheles secretanae* Schweitzer and Feldmann, 2002	Middle Eocene	USA (Southern California)
*Ctenocheles ornatus* Beschin, De Angeli, Checchi, and Zarantonello, 2005	Eocene	Italy
*Ctenocheles hokoensis* Schweitzer and Feldmann, 2001	Late Eocene	USA (Washington)
*Ctenocheles possagnoensis* Busulini and Beschin, 2009	Late Eocene	Italy
*Ctenocheles rupeliensis* (Beurlen, 1939)	Early–Late Oligocene	Hungary, Germany
*Ctenocheles fragilis* Jenkins, 1972	Late Oligocene–Early Miocene	Australia
*Ctenocheles compressus* Jenkins, 1972	Early–Middle Miocene	Australia
*Ctenocheles sclephrops* Jenkins, 1972	Early Miocene	Australia
*Ctenocheles notialis* Feldmann, Schweitzer, and Encinas, 2010	Late Miocene–Early Pliocene	Chile
*Ctenocheles falciformis* Collins in Todd and Collins, 2005	Pliocene–Early Pleistocene	Panama, Costa Rica
